# Behind the scenes of the PRIME intervention: designing a complex intervention to improve malaria care at public health centres in Uganda

**DOI:** 10.3402/gha.v8.29067

**Published:** 2015-10-23

**Authors:** Deborah D. DiLiberto, Sarah G. Staedke, Florence Nankya, Catherine Maiteki-Sebuguzi, Lilian Taaka, Susan Nayiga, Moses R. Kamya, Ane Haaland, Clare I. R. Chandler

**Affiliations:** 1 Department of Medical Statistics, London School of Hygiene & Tropical Medicine, London, UK; 2 Department of Clinical Research, London School of Hygiene & Tropical Medicine, London, UK; 3 Infectious Diseases Research Collaboration, Kampala, Uganda; 4 School of Medicine, Makerere University College of Health Sciences, Kampala, Uganda; 5 Institute of Health and Society, University of Oslo, Oslo, Norway; 6 Department of Global Health & Development, London School of Hygiene & Tropical Medicine, London, UK

**Keywords:** malaria, quality of care, health worker, patient-centred services, health centre management, rapid diagnostic tests, training, complex intervention, intervention design, theory of change

## Abstract

**Background:**

In Uganda, health system challenges limit access to good quality healthcare and contribute to slow progress on malaria control. We developed a complex intervention (PRIME), which was designed to improve quality of care for malaria at public health centres.

**Objective:**

Responding to calls for increased transparency, we describe the PRIME intervention's design process, rationale, and final content and reflect on the choices and challenges encountered during the design of this complex intervention.

**Design:**

To develop the intervention, we followed a multistep approach, including the following: 1) formative research to identify intervention target areas and objectives; 2) prioritization of intervention components; 3) review of relevant evidence; 4) development of intervention components; 5) piloting and refinement of workshop modules; and 6) consolidation of the PRIME intervention theories of change to articulate why and how the intervention was hypothesized to produce desired outcomes. We aimed to develop an intervention that was evidence-based, grounded in theory, and appropriate for the study context; could be evaluated within a randomized controlled trial; and had the potential to be scaled up sustainably.

**Results:**

The process of developing the PRIME intervention package was lengthy and dynamic. The final intervention package consisted of four components: 1) training in fever case management and use of rapid diagnostic tests for malaria (mRDTs); 2) workshops in health centre management; 3) workshops in patient-centred services; and 4) provision of mRDTs and antimalarials when stocks ran low.

**Conclusions:**

The slow and iterative process of intervention design contrasted with the continually shifting study context. We highlight the considerations and choices made at each design stage, discussing elements we included and why, as well as those that were ultimately excluded. Reflection on and reporting of ‘behind the scenes’ accounts of intervention design may improve the design, assessment, and generalizability of complex interventions and their evaluations.

Good quality healthcare for malaria includes accurate diagnosis of suspected malaria cases and provision of prompt, effective treatment with artemisinin combination therapies (ACT) (1); however, in Uganda and elsewhere, health system challenges often limit access to good quality care and contribute to slow progress on malaria control (2–4). In Uganda, good quality care has been described as appropriate clinical processes combined with respectful interpersonal interactions and adequate resources (5). Benefits of providing good quality care include increased demand for services (6–8), improved attendance at health centres (9), better relationships between patients and health workers (10), and increased clinic loyalty (11), potentially producing better health outcomes (12). Interventions are urgently needed to improve the quality of care provided at health centres, increase patient attendance, and ultimately improve health outcomes for malaria and other illnesses (13, 14). However, the optimal approach to improving quality of care is not clear, particularly in low-resource settings (15). Provision of basic training and health education have been tried, but appear to have limited impact, prompting calls for more complex interventions targeting the multidimensional nature of patient treatment seeking (16) and provider practices (17, 18).

For the PRIME trial (19), we developed a complex intervention targeting malaria case management at public health centres in Uganda. Drawing on the available literature (20, 21), we aimed to design an intervention that was evidence-based and grounded in theory, was tailored to our study setting, could be evaluated within a randomized controlled trial, and had the potential to be scaled up sustainably by the Ugandan Ministry of Health. The final PRIME intervention consisted of four components: 1) training in fever case management and use of rapid diagnostic tests for malaria (mRDTs); 2) workshops in health centre management (HCM); 3) workshops in patient-centred services (PCS); and 4) ensuring the supply of mRDTs and artemether–lumefantrine (AL, the first-line ACT for malaria in Uganda). The primary outcome for the evaluation of the PRIME intervention was the prevalence of anaemia (haemoglobin <11.0 g/dL) in individual children under five measured in annual surveys of communities surrounding health centres enrolled in the PRIME trial (19).

Interventions such as PRIME can be considered complex due to their multiple, interacting components, which address multifaceted problems within dynamic systems (22, 23). Responding to calls for more detailed and transparent reporting of intervention components (24–26) and designs (27, 28), here we describe the process of designing the PRIME intervention, including the choices we made and the challenges we faced, and how this shaped the final intervention package.

## Study setting

The PRIME intervention was designed for Tororo, Uganda, an area of high malaria transmission (29). In both health centres and communities, infrastructure is limited. Health centres are generally run by nurses or nursing assistants; many lack electricity, running water, functioning laboratories, and adequate staffing. As a result of system-wide reforms in the 1990s and early 2000s, public healthcare was decentralized and, in theory, provided free of charge (30). Due to frequent stock-outs of essential drugs, including antimalarials, patients were often forced to purchase drugs or go without adequate treatment (31).

## Intervention development

In developing the intervention, we followed a step-wise approach informed by the literature (22, 32, 33), including the following: 1) formative research to identify target areas and refine objectives; 2) prioritization of intervention components; 3) review of relevant evidence to support intervention content; 4) development of intervention components; 5) piloting and refinement; and 6) consolidation of the PRIME intervention theories of change.

### Step 1. Formative research to identify target areas and refine objectives

**Fig. 1:**
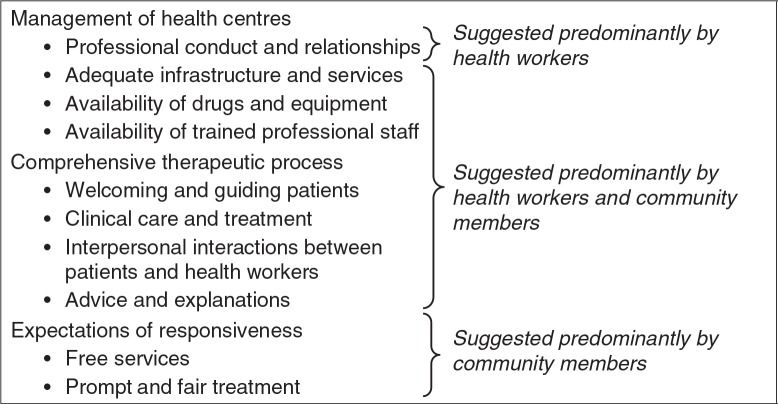
Health workers’ and community members’ aspirations for good quality healthcare.

In 2009–2010, we conducted mixed methods research to characterize the population and local health services using a household survey, situational analysis of government-run health centres, and qualitative assessment of health workers’ and community members’ experiences at health centres (34). Through an iterative thematic analysis, we identified aspirations for good quality care and malaria case management and suggestions of how these might be achieved. Health workers and community members shared ideals of what constituted good care, suggesting that patients might be attracted to attend health centres if quality of care was improved (Fig. 1). However, multiple challenges were identified, including lack of equipment and basic infrastructure, high patient-to-staff ratios, poor health centre management, and stock-outs of antimalarials and other drugs. Social challenges were also identified, including low health worker motivation and difficult relationships between health workers and community members due to lack of trust, language barriers, discriminatory behaviours, and requests for informal payments for services.

**Fig. 2:**
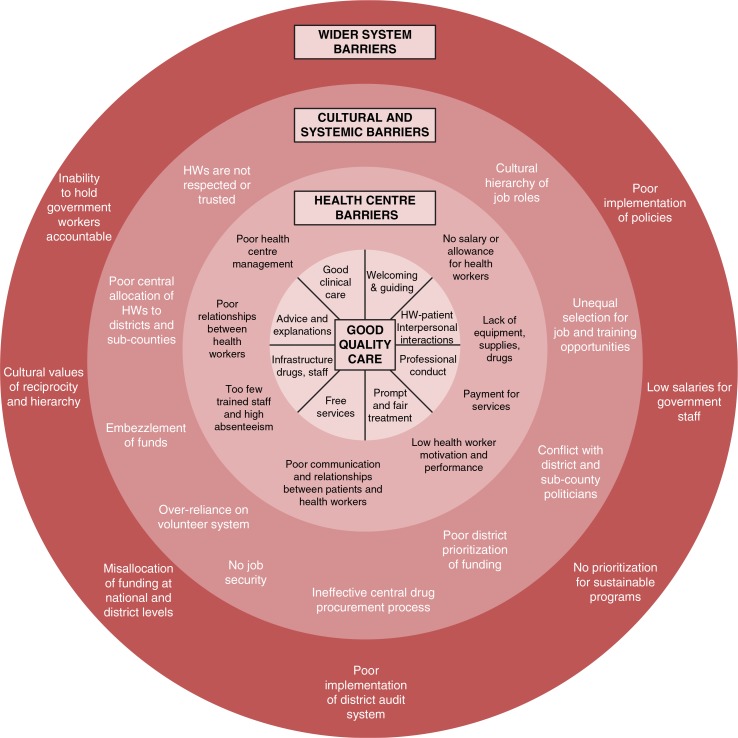
Barriers to providing good quality care at health centres.

We categorized the challenges identified, including health centre factors, cultural and systemic issues, and wider system factors. The results of our analysis identified eight key components of good quality care and corresponding target areas for potential intervention (Fig. 2). Through this process, we differentiated challenges that were amenable to implementation research from those that were beyond the project's scope, thereby reducing a range of complex challenges into a definable set of factors for action at health centres.

### Step 2. Prioritization of intervention components

Prioritizing components to include in the PRIME intervention was an iterative process. We conducted a workshop and follow-up meetings with stakeholders involved in malaria control and child health programmes, including researchers and programme officers at the Ministry of Health, the National Malaria Control Programme, Makerere University, and a local malaria-related non-governmental organization. Together, we reviewed the findings of the formative research and prioritized potential interventions based on stakeholders’ guidance. Overall, stakeholders agreed that we should target malaria case management, patient-centred care, and HCM. However, some of the activities we proposed were deemed beyond the scope of our project. For example, to address staffing shortages and absenteeism, we suggested negotiating with district officials to increase salaries and hire additional staff. We also suggested supplementing the primary healthcare fund – a small cash fund provided to health centre in-charges (often erratically) to pay for essential activities, including transportation of drugs, cleaning services, and necessary supplies. However, district officials were against these propositions, arguing that they would be difficult to administer and sustain. Table 1 outlines further details of these and other activities that were removed from consideration during this process.

**Table 1:** Activities considered but excluded as out of scope for the PRIME intervention

Potential intervention activity	Reasons for consideration drawn from formative research	Reasons not included in the PRIME intervention
Reinstate/supplement the primary healthcare fund	• Insufficient funds to meet daily health centre costs, including transporting drugs, paying for cleaning services, and purchasing supplies • Health workers request payment for services	• Bureaucratically and administratively challenging to implement • Opportunity for misappropriation • Unsustainable after the study period
Fill staffing gaps at health centres in accordance with Ministry of Health guidelines	• Many patients and too few staff • Low motivation of staff due to overburdened workloads • Health centres not fully functional due to insufficient availability of staff • Staff not at recommended levels	• Bureaucratically and politically challenging to implement • Limited availability of health workers nationwide • Requires substantial funding • Unsustainable after the study period
Pay/supplement staff salaries	• Health workers not paid on time or in full • Low motivation of staff due to lack of pay • Time spent in alternative employment activities • Health workers request payment for services	• Bureaucratically and administratively challenging to implement • Not likely to be successful due to national payroll system challenges • Requires substantial funding • Unsustainable after the study period
Implement ICCM through VHTs	• Community medicine distributors/VHTs important source of care, treatment, and referral in the community • Need to determine a sustainable VHT ICCM programme: community sensitization, training, VHT kits, drug supply, supervision	• ICCM and VHT policy under revision and implementation timelines uncertain • Potential challenges with the operationalization of the new policy • Required drug formulations for pneumonia not yet available
Improve the drug supply chain for AL	• Frequent stock-outs of AL and other essential drugs, leading community members to seek care elsewhere • Stock-outs due to challenges with quantification, ordering, storage, district level stock of AL, and numerous logical barriers	• Other programmes already addressing the drug supply chain • Imminent implementation of new ‘push’ system, potential for misalignment • Unlikely to yield results due to challenges at higher levels of the system
Work with district and partners to ensure supply of mRDTs and thermometers	• World Health Organization guidelines for malaria case management, but limited supply of mRDTs to health centres • Thermometers not supplied or available in health centres	• No options for partnering with other stakeholders/partners providing mRDTs and thermometers identified; therefore, they would have to be directly supplied by the PRIME intervention
Implement community sensitization	• Attract patients to health centres by communicating new/improved services using local councillors, social gatherings, word of mouth, mass media, community dialogues	• It was suggested to focus on word of mouth/VHTs to communicate information; however, the VHT programme was not implemented during the study period
Include supervision and coaching as part of HCM modules	• Supervision is described by health workers as ‘fault finding, unsupportive and infrequent’, leading to demotivation	• Weak evidence demonstrating effectiveness of supervision • Challenging logistics of implementing supervision activities
Implement 3-month SOA to complement PCS	• Lack of patient-centred thinking due to low motivation and lack of awareness of how emotions can affect actions and relationships with others	• 3-month activities not aligned with other intervention training packages; therefore revised to weekly activities to fit within four PCS modules

ICCM=integrated community case management; VHT=village health team; AL=artemether–lumefantrine; mRDT=malaria rapid diagnostic test; HCM=health centre management; PCS=patient-centred services; SOA=self-observation activities.

Through this process of prioritization we arrived at four intervention components (Table 2, Supplementary file 1), including the following: 1) training in fever case management and use of mRDTs (FCM); 2) workshops in HCM; 3) workshops in PCS; and 4) supporting the supply of mRDTs and AL.

**Table 2:** PRIME training and workshop modules

Training in FCM		
Aim: To train health workers in use of mRDTs and build clinical skills for managing malaria and other febrile illnesses		

**Barriers addressed**	**Module**	**Topic**

• Poor knowledge of malaria case management • Inadequate/unavailable infrastructure or diagnostic laboratory facilities	Training module	• How to evaluate patients with fever and select patients for mRDT testing • Performing and reading an mRDT • Management of a patient with fever and a positive or negative mRDT • Recognition and referral of patients with severe illness • mRDT storage and monitoring
	Supervision visits	• Observation and feedback on: • Use of mRDTs • Skills in fever case management • Stock management of AL and mRDTs • Recording mRDT results in patient registers

**Workshop in HCM**		

Aim: To develop in-charge health workers’ accountable practices in management of finances, supplies, and health information		

**Barriers addressed**	**Module**	**Topic**

• Poor management of resources by those in charge	HCM 00: Introduction to HCM	• The role of accountability as a health worker
• Low motivation of staff due to poor health centre administration • Under-utilization or lack of appropriate tools to appropriately mange health centres • Low use of records to monitor and manage resources and report to local and district stakeholders	HCM 01: Primary healthcare fund management HCM 02: Drug supply management HCM 03: Health information management	• Budgeting and accounting using the Primary Health Care Fund management tool • Budgeting and accounting – putting it all together • Principles of the drug distribution system • Forms required in drug distribution cycle • The ACT Drug Distribution Assessment Tool • Why quality information matters • The information cycle – from patient to patient

**Workshop in PCS**		

Aim: To improve health workers’ interpersonal communication with patients and other health centre staff and to build consultation skills		

**Barriers addressed**	**Module**	**Topic**

• Lack of patient-centred thinking • Communication problems including language barrier	PCS 00: Introduction to PCS and SOA	• Thinking about my role as a health worker • Introduction to PCS • Introduction to SOA
• Discrimination/preferential treatment of patients • Inappropriate use of volunteers • Poor relationships between staff and communities • Poor patient flow and management	PCS 01: Communication skills part 1 PCS 02: Communication skills part 2 PCS 03: Building a positive work environment PCS 04: Improving the patient visit	• Building rapport • Active listening • Asking good questions • Giving good information • HCM changes • Dealing with stress at work • Communication review • Patient welcome and orientation

mRDT=malaria rapid diagnostic test; HCM=health centre management; PCS=patient-centred services; SOA=self-observation activities.

### Step 3. Review of relevant evidence to support intervention content

We searched for existing training packages in the published and grey literature, both online and in local library collections, prioritizing interventions that had been evaluated and found to be effective in Uganda or similar low-resource contexts.

#### FCM module

For the FCM module, we identified a training package developed by the Joint Uganda Malaria Training Program (JUMP) team, utilizing mRDT training guidelines and job aids adopted by Uganda's Ministry of Health (35). The training consists of lectures and practical sessions, followed by three rounds of support supervision by the JUMP team on-site at health centres (at 1 week, 6 weeks, and 6 months post-training). The training has been shown to improve fever case management and reduce the number of unnecessary antimalarial treatments when implemented in public health centres in Uganda (Hopkins, unpublished observations) (36).

#### HCM and PCS modules

For the HCM and PCS modules, we were unable to identify suitable pre-existing interventions. Although interpersonal interactions between health workers and patients are considered to be central to good quality care (5, 13, 37, 38), the philosophy of PCS is not as prominent in African healthcare (39) as it is elsewhere (40, 41). In addition, most HCM interventions we identified were large-scale and implemented in a top-down format. The Securing Uganda's Right to Essential Medicines (SURE) programme is an example (42; see also Refs. (43–47). Because rigorous evaluations of these programmes have been limited, there was little evidence to inform the PRIME intervention. Thus, we opted to design the HCM and PCS modules ourselves.

Our HCM and PCS modules are based on concepts and resources originally developed by AH for a health-provider communication training model in collaboration with health providers in seven countries in Eastern Europe and Africa and with the Kenya Medical Research Institute/Wellcome Trust Research Programme (Haaland, personal communication, 15 May 2010). The HCM modules were designed to align with existing HCM processes. For the PCS modules, we aimed to strengthen providers’ relationships with patients, colleagues, and the community (38), by reorienting the care-seeking experience towards patients’ aspirations for good quality care (5).

#### Supply of mRDTs and AL

We aimed to align the PRIME supply component with Uganda's existing supply system and the SURE programme (42). However, while we were developing the intervention, Uganda's National Medical Stores (NMS) distribution system changed from a ‘pull’ system, in which drugs were ordered by health centres, to a ‘push’ system, with regular delivery of a pre-determined package of drugs, requiring us to revise the PRIME supply component. We identified an existing health worker to act as a liaison, who was responsible for gathering stock information from health centres and facilitating delivery of mRDTs and AL from PRIME when the NMS supply was inadequate or failed. The SURE programme, introduced in 2009, aimed to gather drug stock information and minimize stock-outs through supervision visits. The PRIME intervention utilized SURE's pharmaceutical management information system forms and procedures.

### Step 4. Development of intervention components: HCM and PCS modules

To develop the HCM and PCS modules, we reviewed evidence on successful intervention activities; translated evidence into content; incorporated behaviour change theory, adult learning cycles, and learning activities; and created workshop manuals.

#### Reviewing evidence on successful intervention activities

We reviewed the literature to identify activities targeting health worker communication and interpersonal relationships, patient satisfaction, health worker supervision and coaching, and management of health centres. We focused on low-cost and low-resource interventions, prioritizing interventions that had been successfully implemented and evaluated. The review methods are described elsewhere (Chandler, unpublished observations).

Several activities have been shown to improve communication between health workers and community members, producing a positive effect on patient satisfaction and health outcomes. These activities include enabling clinicians to give patients time to talk during a consultation by asking good questions (48) and employing active listening (49, 50) to elicit better information from patients (51). Activities to build rapport and support emotional care by reassuring patients (52) have also been shown to facilitate patients’ therapeutic reactions (53–55). Likewise, activities promoting ‘positive communication’ may improve teamwork by recognizing how personal circumstances and work environment affects emotions and communication (52, 56). Activities to improve relationships between health workers include building self-awareness and constructive communication through vignettes, which are used to identify and resolve sources of conflict (57). Notably, of these activities, only ‘time to talk’ was drawn from a low-income setting.

Activities shown to improve patient satisfaction with experiences at health centres include greeting patients (58) and guiding patients through the health centre (46). Interventions promoting supervision and coaching were also identified, although evidence that these activities change provider performance was weak (59, 60). We also considered health worker performance management programmes, including the SURE programme and the Uganda Malaria Surveillance Programme's (UMSP) Continuous Quality Improvement Project, which demonstrated that providing health status reports and regular supervision with constructive feedback improved health worker performance (Mpimbaza, personal communication, 10 June 2010). However, the UMSP activities had not been systematically evaluated. Thus, we were forced to weigh the available evidence and decide which activities best informed the design of our intervention package. We ultimately chose not to include coaching or supervision due to the concerns about sustainability, both during the trial and if scaled up, and the limited evidence base supporting coaching and supervision in our setting (61, 62).

#### Developing intervention content

For drug supply management, we drew on the literature to develop the ACT Drug Distribution Assessment Tool, a one-page tool to support health workers in resolving everyday distribution bottlenecks that are not tracked in standard monitoring tools, but are often the cause of health centre drug stock-outs (63). For financial management, we developed the Primary Health Care Fund Budgeting and Accounting Tool, a one-page tool to assist health workers with managing the health centre primary healthcare fund.

For the PCS modules, we adapted activities to improve health worker communication developed mainly in high-income settings to our study setting by using local cultural and social references drawn from our formative research. We deconstructed concepts contained in activities such as *giving time to talk*, *building rapport and emotional care*, and *self-awareness* and reconstituted these in forms and definitions meaningful to the study context. Thus, activities maintained their intended purpose but were communicated using scenarios and discussion points relevant to health workers’ everyday experiences.

#### Incorporating behaviour change theory

The HCM and PCS modules are underpinned by behaviour change theory to initiate the intended pathway of effect. Both modules aimed to build a supportive community of practice. The Communities of Practice behaviour change theory posits a cyclical process of change, where individuals’ frames of reference are transformed through participation in a community of peers, and their participation in turn transforms the community (64). This process serves to create an ‘informal curriculum’ for health workers in addition to the existing overarching core curricula (65). Through this process, learners engage with other community members and reflect critically on their practice through a social process of individual and collective learning (66).

The theory of Communities of Practice resonated in our setting where many health workers learn primarily on the job. Likewise, our setting lacks many external motivators that have been shown to promote health worker performance, such as financial incentives, constructive supervision, professional accreditation, and opportunities for promotion (67–71). Therefore, we sought to balance the limitations of the context with the opportunity to stimulate health workers’ internal motivations for providing good quality care (72), which included the desire to be viewed as professional, to be respected by colleagues and community members, and to be valued for providing good healthcare services (34). We theorized that, as health workers built, demonstrated, and received positive feedback on their clinical, interpersonal, and managerial skills, the social processes emerging from participation in the community of practice would help them to develop their professional identity and sustain positive skills and behaviours (73).

#### Incorporating an adult learning cycle and learning activities

The HCM and PCS modules were designed as interactive weekly 3-hour workshops to promote group learning, contributing to the development of a community of practice. The structure was designed to allow time to reflect and practice skills in between workshops and to get feedback at subsequent workshops. Small groups of health workers were selected to enhance participation and encourage peer support in the future. The workshops were led by three members of the PRIME research team, who had medical backgrounds but little experience in interactive training methods, as is the norm in Uganda (74).

The workshops were framed as continuing professional development with interactive learning activities which have been shown to improve health worker knowledge, skills, attitudes, and behaviours leading to improved patient outcomes (75–77). The workshops were structured as a six-step adult learning cycle drawn from Kolb's (78) experiential learning theory, which includes four stages: experience, reflection, conceptualization, and planning; and from Knowles’ (79) theory of adult learning, which asserts that adults must first establish why they should learn something before proceeding to acquiring new knowledge. The six steps involve developing a ‘need to know’, individual reflection, conceptualization, experimentation, group reflection, and planning. To activate this learning cycle, the workshops employ a variety of participatory learning methods drawn from training modules in similar contexts (Supplementary file 2) (Haaland, personal communication, 15 May 2010) (37, 48).

The PCS module also included weekly self-observation activities (SOA) that aimed to stimulate learners’ purposeful critical analysis of their knowledge and experience (80), enabling them to engage and deal with their emotions (81) and develop appreciation and respect for others (82). Semi-structured SOAs followed by feedback in groups provided opportunities for both individual learning and change as a community (66). The SOAs were adapted from tasks designed and tested in a number of other healthcare settings (Haaland, personal communication, 15 May 2010) (48).

#### Creation of Workshop Manuals

For each HCM and PCS workshop, we created corresponding trainer and learner manuals – 18 in total (Supplementary file 2). We contracted with an experienced public health consulting firm (83) to fine-tune the learning activities and typeset the manuals. This was a collaborative effort, requiring significant input on a new layer of design considerations, including the colours and fonts that would best communicate the ethos of the workshops and how pictures and layout of activities could support learning retention. We also considered how the trainer instructions would encourage active facilitation but also support trainers in drawing out learners’ reflections and experiences.

### Step 5. Piloting and refinement: HCM and PCS modules

We conducted two rounds of piloting the HCM and PCS modules with 10 health workers from outside of the study area. We administered questionnaires to learners and trainers, gathered daily feedback from trainers and the piloting team, and conducted focus group discussions with participants at the end of the modules. The piloting evaluated the relevance and applicability of the learning objectives and content, as well as the delivery of the training (84). The piloting proved to be an invaluable exercise, unexpectedly revealing that the learning capacity of our intended learners was not in line with our expectations. Whereas the six-step learning process and interactive activities appeared to support learning, some of the module concepts and language were too advanced, requiring us to readjust our expectations of how these concepts could be feasibly introduced. The trainers, who had more experience with didactic approaches, also reported challenges with the interactive format of the manuals. Thus, we revised the modules, aiming to ‘hit the mark’ with our intended learners by simplifying the language, reducing the number of new concepts and learning objectives per module, including more interactive activities, and revising the prompts and instructions throughout the trainers’ manuals. See Table 3 for examples of revisions made. The second round of piloting indicated that the revised modules did meet our intended objectives. However, the piloting and subsequent revisions added significant and unexpected delays to the design process. The final learning objectives are in Supplementary file 1, and final versions of the modules can be found online (85).

**Table 3:** Example of revisions made to the PCS and HCM modules as a result of piloting

Description of revision made	Reason for revision	Example of revision made
Reduced the total number of objectives across the modules so that only one or two new concepts were introduced per module	The total number of learning objectives and amount of content was ambitious for the 3-hour module format. Learning was best taken up when there were only one or two concepts per module.	• Concepts for improving communication with patients were introduced over two modules with two concepts per module: • PCS 01: Communication Skills Part 1 introduced building rapport and active listening • PCS 02: Communication Skills Part 2 introduced asking good questions and giving good information
Simplified language and revised learning objectives to only introduce only one new word per module	Overall, the language needed to be reduced to meet the education level of the learners New words required time and expertise to introduce and be taken up by learners.	• Reduced number of new words (such as *building rapport*, *triage*, *open/closed questions*, or *automatic emotional responses*) to one or two per module
Revised learning objectives to include more group work activities	Learners responded well to group work activities, were more engaged with each other, and retained more learning points, compared to didactic teaching activities. For example, learners struggled to understand and perform calculations required for drug supply management when these were taught didactically.	• Revised learning objective for drug supply management to ‘Accurately complete the forms required in the drug distribution system’. Calculations for the forms were completed as group work, and more information was provided in the learners’ manual for later reference when completing forms at the health centre.
Rephrased objectives with abstract concepts into simpler ideas communicated with activities or games	Abstract concepts took a long time to introduce and give adequate examples; learners understood concepts better when they had an example or activity to describe the concept.	• Learning objective on appreciating barriers to attending the health centre, both logistical (transportation, time, etc.) and emotional (anxiety, confusion), was introduced using a maze activity to demonstrate how these barriers prevent access to health services.

HCM=health centre management; PCS=patient-centred services.

### Step 6. Consolidation of the PRIME intervention theories of change

**Fig. 3:**
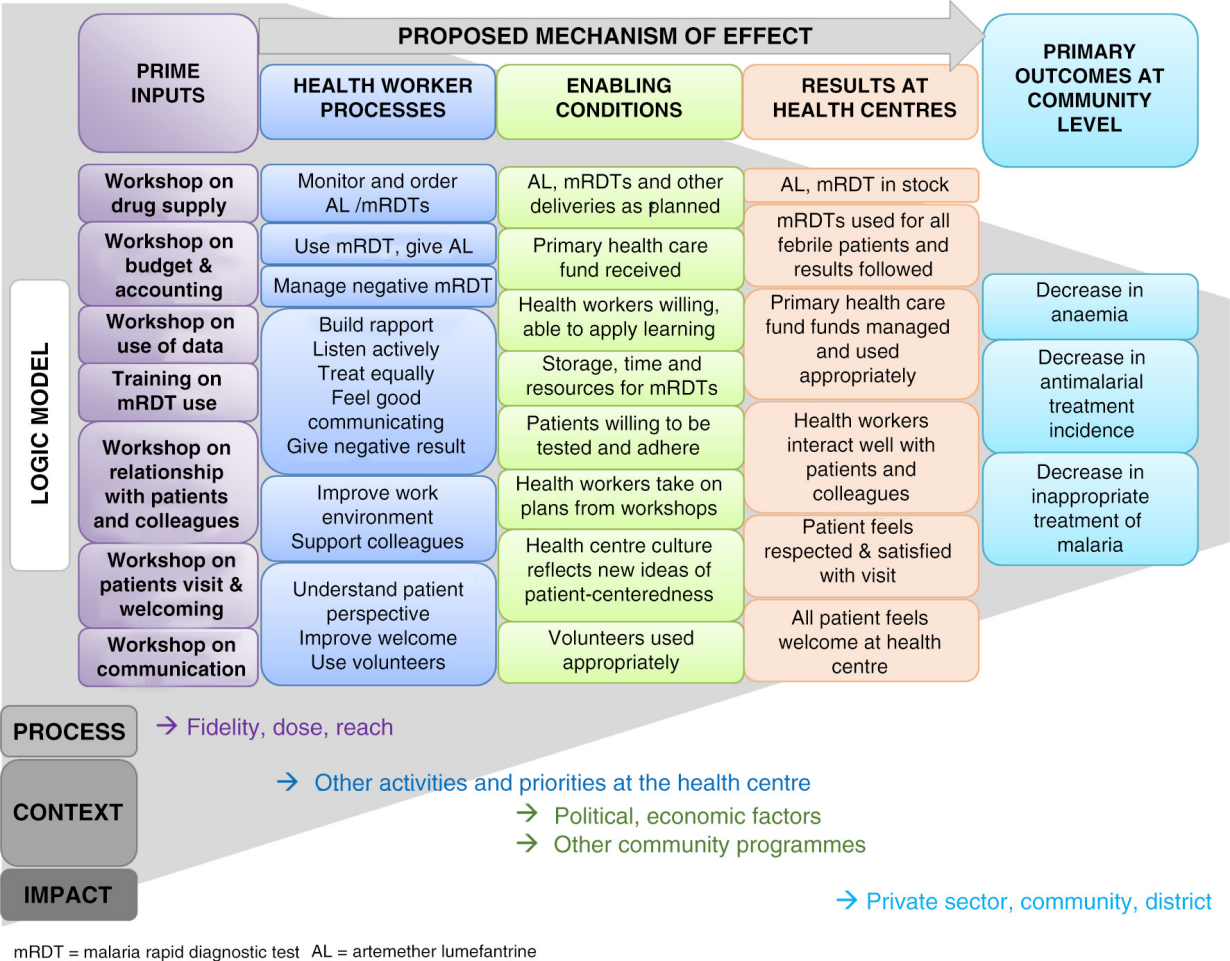
PRIME intervention programme theory and logic model.

**Fig. 4:**
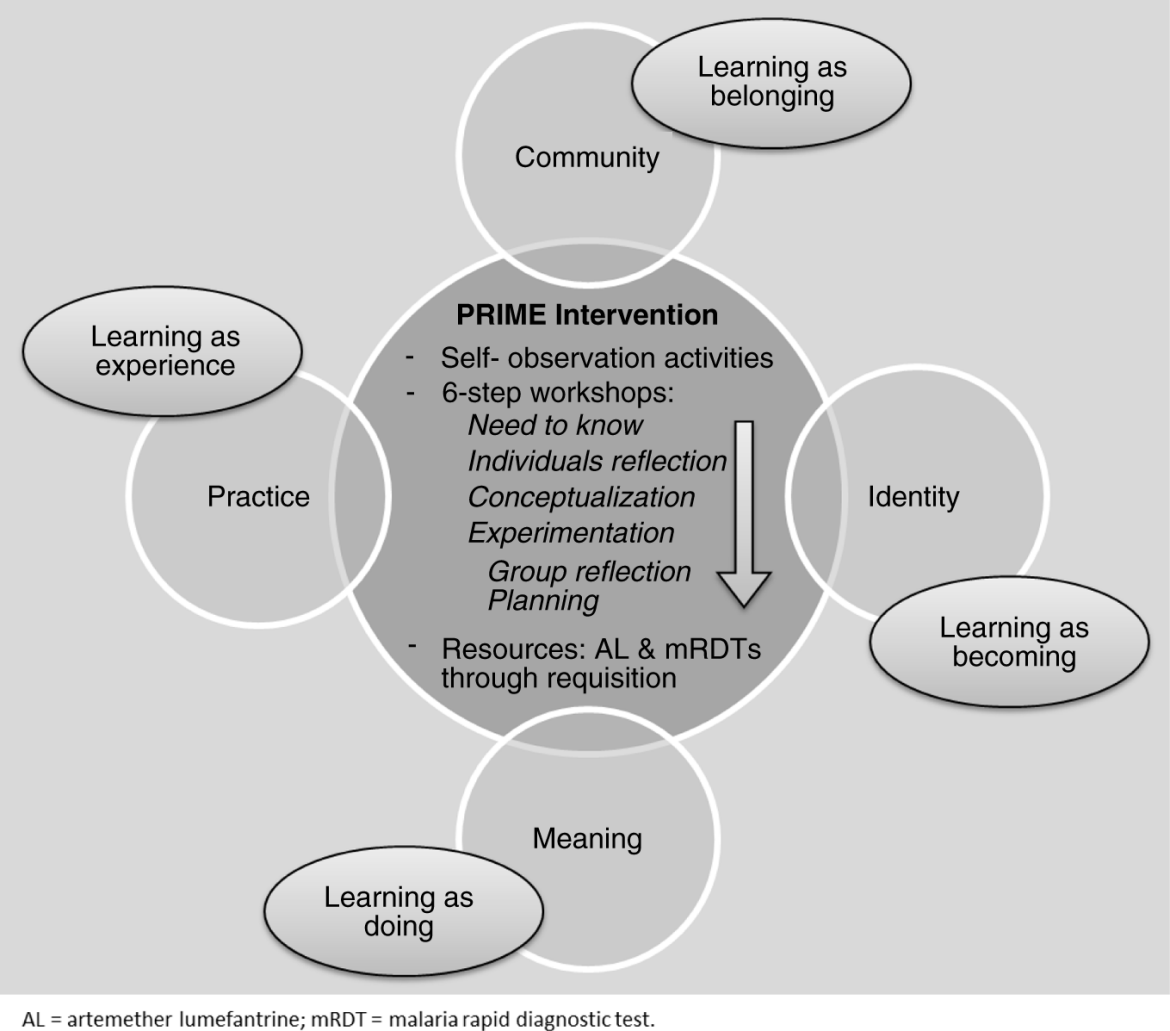
PRIME implementation theory. (Adapted and reproduced with permission from Ref. 39.)

Drawing on complex intervention design and evaluation guidance (22, 86–88), we articulated two complementary intervention theories – a programme theory and an implementation theory. These theories make explicit how and why we hypothesized the PRIME intervention components would combine to produce desired outcomes (89). The programme theory, represented in a logic model (Fig. 3), describes *why* the four intervention components are anticipated to produce specific outcomes. It hypothesizes that an intervention addressing the barriers to providing good quality care for malaria and febrile illnesses will improve appropriate malaria case management and patient satisfaction, leading to repeat attendance at health centres, and ultimately, improved health outcomes in community children. The implementation theory (Fig. 4) articulates *how* the intervention will stimulate behaviour change. It hypothesizes that a learning process stimulating health workers’ cognitive, emotional, and social learning processes through interactive workshops reinforced within a community of practice will lead to immediate and sustained change in health worker motivation, behaviour, and practice for providing good quality care.

## Discussion

We designed a complex intervention targeting delivery of care for malaria at public health centres in Uganda (19, 90). Informed by best practice, we aimed to develop an intervention that was evidence-based, grounded in theory, and appropriate for our study setting using a systematic approach. In the process, we learned several important lessons related to the scope of the intervention and necessary compromises, the tension between static interventions and dynamic contexts, and the challenges of rigorously designing a behaviour change intervention for low-resource settings. By transparently reporting our ‘behind the scenes’ accounts, we hope to inform the design and content of future complex interventions.

Our formative research identified several challenges to providing good quality care at different levels of the health system. Many of these challenges were interpreted to be rooted in wider health system norms that prioritize technical skills and technologies over a patient-centred approach to care. Likewise, our context is characterized by ineffective political systems and a deeply embedded hierarchical structure that perpetuates power imbalances throughout the health system (5). In an attempt to define factors for action at the health centre level, we found it necessary to bracket out much of the complexity and the political–economic reality underlying health service provision in Uganda. As a result, we focused on intervention components that had the highest likelihood of success and buy-in from stakeholders within the constraints of a focused project, which others have noted as a critical factor for success when designing health service interventions (91). However, this choice meant that the deeper social, political, and economic challenges that underlie poor healthcare quality and lack of progress on malaria remained unaddressed by our intervention (92). Rather than ignore these challenges as being out of the scope of the intervention design process, engaging with them was required to situate the intervention within the wider health system context and to provide deeper insight into how the intervention components might operate within this system. Recognizing that interventions are a part of complex health systems (23), we urge intervention designers to consider and report on the process of negotiating wider social, political, and economic realities and how this influenced intervention content and design.

The slow and iterative process of intervention design contrasted with the continually shifting study context. During the intervention design process, which took almost 1 year, several changes occurred in the study context that had significant impacts on the intervention. The integration of the SURE programme and implementation of NMS's push delivery system required a reconceptualization of the HCM modules and the supply component. A policy introduced by the District Health Office to remove untrained volunteers from health centres required an adaptation of the PCS module to suit other authorized support staff. This ever-changing context created a ‘moving target’ with which to align the intervention and contrasted with the need to develop standardized content suitable for evaluation in a cluster-randomized trial. To accommodate this situation, the modules were designed as a structured framework complemented by reflective learning activities to engage with learners’ everyday experiences. In this way, the structure of the modules was standardized and reproducible, but the learning points could be adapted to the local context (93). While the challenge of implementing and evaluating static interventions in dynamic contexts has been considered (94–97), we encountered similar tensions during intervention design. To resolve these issues, flexibility and responsiveness were needed. Although this required additional investments of time and resources, we found this was essential to designing an intervention appropriate for our study setting.

Developing the PRIME intervention required a diversity of expertise, including clinicians, social scientists, epidemiologists, health workers, project managers, and training consultants. Team members approached the design of the intervention from different epistemological and disciplinary backgrounds. Developing the logic model suited the positivist perspective favouring a representation of the intervention as discrete components leading to predefined measurable outcomes. The process of developing the logic model provided an opportunity for the team to share and consolidate ideas and emerged as a convenient communication tool. However, the static nature of the model did not adequately capture the way we expected change to occur, recognizing that change processes would be dynamic, emergent, and contingent on links between the intervention, individuals, and society (98). By utilizing both text and visual models as part of our intervention theories, we endeavoured to articulate a specific intervention theory while acknowledging that the intervention would be enacted in a dynamic context, which would create many unique change processes, both intended and unintended. Our different disciplinary perspectives also led us to engage with questions of what the intervention ‘is’ – for example, rather than simply a composition of training materials and events, we began to conceive it as a series of interactions embedded in social relationships through which its meaning would emerge. This raised the possibility that the meaning of the intervention could be constructed differently by different actors, which was important to capture in our evaluation activities. Our experience concurs that an interdisciplinary approach appears to be essential for making meaningful progress towards improving population health (99); however, it should be recognized that this approach is time- and resource-intensive (91, 100, 101), requiring concerted effort to align perspectives into a shared understanding of the intervention (102).

Our experience designing the PRIME intervention reflected a process that is more interactive and demanding than the available evidence and theory suggest (20). While the literature guiding intervention design is expanding (91, 103), few authors discuss the construction process we found necessary to reach the final intervention package. The importance of reporting ‘insider accounts’ of intervention implementation and evaluation activities to better interpret trial outcomes has been noted (104, 105). We argue that this same reflective and transparent reporting practice should apply to intervention design. Guidelines for reporting complex intervention content ask authors to describe the reasons for selecting intervention components, which may include ‘experience of or evidence on the suitability of the component to achieve the intended change process’ (4: (106). Our experiences revealed manifold reasons influencing the processes through which intervention content was considered, shaped, and integrated (or discarded), in light of research aims, available evidence, and resource constraints. Sharing accounts of activities that were considered but omitted, and why these decisions were made, may be as informative as descriptions of final intervention packages. Thus, we argue that describing these behind-the-scenes accounts of the intervention design process should be considered a key ‘experience’ included in guidelines for reporting intervention content and their evaluations. A reflective and transparent reporting of the design process may promote assessments of the intervention's internal validity, facilitate interpretation and generalizability of results, and inform future interventions. As complex interventions gain momentum in healthcare, guidelines for developing interventions and reporting on the design process will need to evolve, consistent with current debates of how complex interventions should be conceptualized and evaluated (23, 98, 107).

## Supplementary Material

Behind the scenes of the PRIME intervention: designing a complex intervention to improve malaria care at public health centres in Uganda
