# Supplementary material for: Behind the scenes of the PRIME intervention: designing a complex intervention to improve malaria care at public health centres in Uganda
Source: Glob Health Action. 2015 Oct 23;8:10.3402/gha.v8.29067. doi: 10.3402/gha.v8.29067 (PMC4620687; doi:10.3402/gha.v8.29067)
Supplement: Behind the scenes of the PRIME intervention: designing a complex intervention to improve malaria care at public health centres in Uganda [file GHA-8-29067-s001.pdf]

Supplementary material

Supplementary material 1: PRIME intervention components

Training in Fever case management		
**Aim:** To train health workers in use of mRDTs and build clinical skills for managing malaria and other febrile illnesses.		
**Barriers targeted:**<br>• Poor knowledge of malaria case management<br>• Inadequate/unavailable infrastructure or diagnostic laboratory facilities		
**Module**	**Topic**	**Learning outcomes**<br>By the end of this module, participants should be able to:
**Fever case management training module**	How to evaluate patients with fever and select patients for Rapid Diagnostic testing	• Describe fever and explain how this symptom is important in selecting a patient for mRDT testing<br>• List signs of severe illness in a patient with fever<br>• Outline important questions to ask when taking a history from a patient with fever<br>• Describe how to carry out a physical examination of a patient with fever<br>• Describe how to select a patient for mRDT testing based on the history and physical examination
	Performing and reading a mRDT	• Describe a mRDT and how it works<br>• Perform a mRDT correctly and safely<br>• Read a mRDT accurately and record the result as positive or negative<br>• List some important tips for using mRDTs<br>• Describe safe handling of blood and sharps
	Management of a patient with fever and a positive mRDT	• Explain the meaning of a positive mRDT result in a patient with fever<br>• Describe how to treat a patient with fever and a positive mRDT<br>• Outline supportive treatments for a patient with fever and positive mRDT
	Management of a patient with fever and a negative mRDT	• Outline the benefits of treating patients on the basis of mRDT results<br>• Explain the meaning of a negative mRDT in a patient with fever<br>• Describe the management of a patient with fever but a negative mRDT<br>• List and describe the management of some common non-malaria febrile illnesses
	Recognition and referral of patients with severe illness	• List danger symptoms and signs of severe illness<br>• Outline the steps to refer severely ill patients to higher level health facilities<br>• Describe pre-referral treatments that may be given to severely ill patients before transfer to a higher level health centre
	Patient education	• List 5 good communication skills<br>• Outline important messages to give to a patient/caregiver to encourage adherence to treatment<br>• Outline important messages to give a patient/caregiver on symptoms that indicate a need to return to for further care<br>• Outline important messages to give a patient/caregiver on malaria prevention
	mRDT storage and monitoring	• Describe the proper storage conditions for mRDTs<br>• Explain how to monitor mRDT expiry dates with the first expiry, first out' principle

**Fever case management supervision visits**	First supervision visit: within 1 week of training	• Observe and mentor health workers on how to:<br>• Manage fever patients with positive or negative results<br>• Set the working area<br>• Record mRDT results in the registers<br>• Assess and replenish stocks of AL and mRDTs
	Follow-up supervision visit: 6 weeks and 6 months after initial training	• Observe and mentor how health workers are:<br>• Evaluating patients with fever.<br>• Performing the mRDTs 6 weeks after the training.<br>• Reading the results<br>• Managing the results of mRDTs<br>• Recording the results and treating patients<br>• Identify and address weakness and challenges of mRDT use in fever patient management.
**Workshop in Health Centre Management**		
**Aim:** To develop in-charge health workers' accountable practices in management of finances, supplies, and health information.		
**Barriers targeted:**<br>• Poor management of resources by in-charges at health centres<br>• Low motivation of staff due to poor health centre administration<br>• Under-utilization or lack of appropriate tools to appropriately mange health centres<br>• Low use of health centre records to monitor and manage health centre resources and report to local and district stakeholders		
**Module**	**Topic**	**Learning outcomes**<br>By the end of this module, participants should be able to:
**HCM 00 Introduction to HCM**	Accountability	• Understand the meaning and role of accountability for in-charges<br>• Recognize how being accountable impacts on others' perception of in-charges<br>• Describe the role of accountability in good health centre management
**HCM 01 Primary Health Care Fund management**	Budgeting and accounting using the Primary Health Care Fund management tool	• Describe the Ministry of Health policy for Primary Health Care Funds for health centres II/IIIs<br>• Understand the rationale for training in budgeting, accounting, and Primary Health Care Fund management<br>• Describe how the health centre uses its Primary Health Care Funds<br>• Recognize how in-charges can build trust and accountability in their roles through good Primary Health Care Fund management<br>• Describe the principles of budgeting and accounting<br>• Develop and apply budgeting and accounting skills using the Primary Health Care Fund Management Tool
	Budgeting and accounting -- putting it all together	• Describe the importance and benefit of budgeting and accounting for the Primary Health Care Fund<br>• Understand how budgeting and accounting contributes to showing accountability and skill as an in-charge<br>• Plan and commit to completing the Primary Health Care Fund Management tool regularly at their health centres
**HCM 02 Drug Supply Management**	Principles of the drug distribution system	• Describe the main components of the drug distribution system<br>• Be motivated to actively participate in and keep the drug distribution system on track
	Forms required in drug distribution cycle	• Describe the purpose and benefit of completing forms required in the drug distribution system including the Out-Patient Department register, Stock-card (Form 015), Order Form (Form

		085)<br>• Accurately complete the forms required in the drug distribution system<br>• Put in place a plan for completing the forms regularly at the health centre
	The ACT Drug Distribution Assessment Tool	• Identify issues that prevent drugs from reaching the health centre<br>• Identify and implement solutions to the issues that prevent drugs from reaching the health centre<br>• Be motivated to complete the ACT Drug Distribution Assessment Tool regularly
**HCM 03 Health Information Management**	Why quality information matters	• Understand why we collect patient information
	The information cycle -- from patient to patient	• Understand how collecting information can beneficial to the health centre (drug quantification, predicting future needs)<br>• Understand how collecting information improves patient management

Workshop in Patient-Centred Services

Aim: To improve health workers' interpersonal communication with patients and other health centre staff and to build consultation skills.

Barriers targeted:

- Lack of patient-centred thinking
- Communication problems including language barrier
- Discrimination/ preferential treatment of patients
- Inappropriate use of volunteers
- Poor relationships between staff and communities
- Poor patient flow and management

**Module**	**Topic**	**Learning outcomes**<br>By the end of this module, participants should be able to:
**PCS 00 Introduction to PCS and Self-Observation Activities**	Thinking about my role as a health worker	• Identify one's own motivations for work.
	Introduction to PCS	• Understand the meaning and importance of providing patient centred services.
	Introduction to SOAs	• Start developing self-awareness through self-observation activities.
**PCS 01 Communication Skills Part 1**	Building Rapport	• Recognise the impact of non-verbal and verbal behaviour on the patient and consultation outcome.<br>• Strengthen non-verbal and verbal skills in building rapport.<br>• Recognise that we think of different people in different ways, and this affects how we behave towards them.<br>• Understand that respect is a core value for how we can put patients at ease.<br>• Strengthen skills to show respect to patients.
	Active listening	• Strengthen skills in self-reflection.<br>• Strengthen non-verbal and verbal skills in active listening.<br>• Recognise the consequences of listening well, and less well, on the patient and consultation outcome.<br>• Identify ways to listen actively in spite of busy work environments.
**PCS 02 Communication Skills Part 2**	Asking good questions	• Understand the importance of getting good information.<br>• Be aware of the way and consequences of how they ask questions.

		• Know how to formulate open questions.<br>• Ask questions without showing judgement.
	Giving good information	• Understand the importance of giving good information.<br>• Be aware of the way and consequences of how they give information.<br>• Know how to give good information to patients.<br>• Understand how to empower patients to follow advice.
**PCS 03 Building a positive work environment**	Health Centre Management Changes	• Recognise their challenges at work.<br>• Know about planned Health Centre Management changes.<br>• Know their role in Health Centre Management changes.
	Dealing with stress at work	• To recognise stress by how we feel and behave.<br>• To understand the effect of automatic reactions on us and others.<br>• To know how to 'step back' and stop automatic reactions.<br>• To carry a picture of best practice in dealing with difficult patients and situations.
**PCS 04 Improving the Patient Visit**	Communication Review	• Become aware of ways to invite their patients and colleagues to co-operate and the impact of doing this.
	Patient Welcome and Orientation	• Recognise that we all have different perspectives, including as health workers and patients.<br>• Put themselves into the shoes of a patient approaching a health centre as an organisation with unspoken 'rules'.<br>• Explore reasons why patients have to wait long, and develop strategies that meet health workers' as well as patients' needs better.<br>• Implement strategies to improve the welcome of patients at health centres.<br>• Implement strategies to improve the orientation of patients at health centres.<br>• Implement strategies to ensure patients are seen fairly.

PCS = patient-centred services; HCM = health centre management; mRDT = malaria rapid diagnostic test.

Supplementary material 2: PRIME Intervention Learning & Design Principles

The PRIME Intervention Learning & Design Principles

The structure, design and layout of the Patient-Centred Services (PCS) and Health Centre Management (HCM) manuals was designed to assist trainers with facilitating the interactive group-style learning and to assist learners with actively engaging with the trainer and the manuals including:

1. Formatting and typeset principles
2. 6-step adult learning cycle
3. Participatory training activities
4. Icons to facilitate use of manuals

1. Formatting and typeset principles

The learner manuals were designed as workbooks with space allocated for answering questions posed by the trainer, completing activities, taking notes, and recording reflections. A workbook-style manual was created to improve engagement and retention of learning and provides a reference for learners' responses for reference after the training.

The visual format of the manuals was designed to communicate a positive and motivating learning process. The page layout, colour schemes, and fonts were designed to be inviting, friendly, and visually appealing. The manual design is also suitable for printing in greyscale on standard A4 paper in order to provide a lower cost option for reproducing the training manuals on a large scale.

A consistent structure for title page, introductions, training agenda, description of learning activities, learning summary boxes, and conclusion were used throughout each manual to create a professional product which intended to be easily taken-up by trainers in Uganda, and potentially elsewhere.

2. PRIME 6-step adult learning cycle

Step 1: Need to know
In order for the learner to become engaged, the individual must understand why they should participate in the learning, building on their own experiences.

Step 2: Individual reflection
Deep learning involves reflecting on experiences, analysing patterns of behaviour and identifying consequences. The team followed the appreciative inquiry approach to facilitation which intends to identify and focus on positive behaviours and build on these in moving forward.

Step 3: Conceptualisation
If learners can identify a pattern in their experiences (of either 'old' behaviour or 'new' ideas from learning) then the learner can form a generalisation and a set of concepts to define the situation.

Step 4: Experimentation
Once solutions have been identified in theory to problems identified from experience, these solutions need to be tested in practice.

Step 5: Group reflection
Learning is not an isolated experience but a collective process involving cultural formation and shifts in perspectives of groups.

Step 6: Planning
After learning something new, it is necessary to complete the cycle by identifying how the learning can be applied to the learner's job, to solve their problems, including setting commitments for future learning and changes and mechanisms to monitor and support peers in achieving these commitments.

3. Participatory learning activities

The following outlines the participatory learning activities in the PCS and HCM modules. Activities such as group presentation and group discussion are used during the 'Principles' step of the learning cycle. Skill-based activities such as role-play and demonstrations are used during the 'Conceptualisation' and 'Experimentation' steps of the learning cycle to practice and discuss new skills. Group participation activities such as group discussion and small group work are used during the 'Group reflection' and 'Planning' steps of the learning cycle to foster teamwork and a community of practice. Throughout the modules, interactive activities such as buzzing, question and answer, flip chart, the parking lot, and energisers are used to keep learners active and engaged.

Drawings are included to communicate practices of good quality care to health workers (1) and act as a reminder when text may be daunting to read. We applied principles of picture design following (2) to create a visual representation of the main learning outcome for each PCS and HCM module.

Group presentation by the trainer is used to present theory or concepts to the group in plenary. Active listening and participation is encouraged through asking questions to the group, placing information on the flip chart, reading aloud from the Learner Manual, and writing in the Learner Manual.

Group discussion is used to gather information, thoughts, and responses to questions or activities from the group. By discussing with others in a 'safe space', participants are able to justify their own learning experiences and learn from the experiences of others. This helps participants to feel that their new ideas, skills, or behaviours are acceptable to their peers, making them more likely to be applied in everyday situations.

Small group work is used to increase understanding of a topic or concept, discuss participant experiences, and to practice new skills. Constructive feedback is encouraged to improve group dynamics and promote teamwork, build participants' confidence in their new skills or behaviours, and provide a foundation for group work or discussions in the workplace.

Role play a dramatic acting of a scenario or behaviour and is used to help discuss sensitive issues which can be difficult to address in plenary and to assess whether participants have acquired the knowledge and are able to apply it in practice.

Demonstrations are used by the trainer or learner to show how to perform a skill correctly. Demonstrations are an effective way of teaching skills that are hard to describe in words or involve body actions, for example communication skills, or for practicing how to complete tasks correctly, for example completing routine forms.

Buzzing is used as a quick method to have participants, usually pairs or up to groups of four, discuss together and then write responses in their Learner Manual or feedback responses to the group. Buzzing encourages participation and active learning with peers.

Questions and answers are used to encourage participation and active learning by assessing learners' level of knowledge or learning on a topic or concept or to gain learners' feedback. Trainers encourage learners to think of and provide their response by 'giving time' to participants after asking questions.

Flipchart papers are used to present information or record responses provided by learners. Flipchart headings or content are prepared ahead of the training to save time and help keep the trainer on-track.

The Parking Lot is a blank flipchart paper used to acknowledge and record learners' questions or observations, which do not relate to the topic at hand but may be addressed in a previous or subsequent topic or module. Trainers 'park' the question and address anything outstanding by the end of the training or by the next training session to show learners that their contributions are values and appreciated.

Energisers are included to combat restlessness or boredom by introducing an activity that encourages learners to move and become more alert. Short energisers such as a two-minute exercise or dance are encouraged.

4. Icons to facilitate use of manuals

The following icons are used throughout the PCS and HCM manuals to signpost key activities to trainers and learners. Icons were developed and used throughout the manuals to signpost key activities. The icons are simple black and white line drawings and were placed in the margins of the manuals for easy identification.

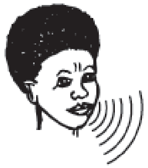

Talking Point: This icon indicates text for the trainer to speak to the participants. These are the sections of text you should be familiar with and perhaps use a highlighter pen to emphasize the most important sections of the text you want to present to the participants.

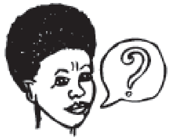

Asking point: This icon indicates text for the trainer to ask to the participants. Try to use the questions written in the training manual – these are 'open-ended' questions that allow participants to think of and give a 'rich' response, rather than a quick 'yes or no' response.

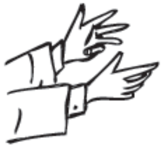

Activity: This symbol indicates that an activity is to take place and should remind you that materials or special instructions may be needed.

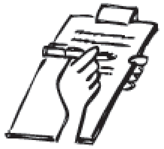

Summary box: This icon indicates a box with a summary has been included in the manual, for reference for trainers and learners.

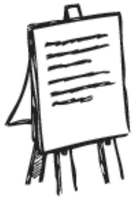

Flipchart: This icon is used to show you when you should be writing or placing items on the flipchart.

References

1. Houts PS, Doak CC, Doak LG, Loscalzo MJ. The role of pictures in improving health communication: a review of research on attention, comprehension, recall, and adherence. Patient Educ Couns. 2006;61(2):173–90.

2. Haaland A. Reporting with Pictures. A concept paper for researchers and health policy decision-makers [Internet]. Geneva: UNDP/World Bank/WHO Special Programme for Research and Training in Tropical Diseases; 2001 [cited 2014 Mar 15]. Available from: http://www.who.int/tdr/publications/documents/pictures.pdf
